# *Ranbp2* haploinsufficiency mediates distinct cellular and biochemical phenotypes in brain and retinal dopaminergic and glia cells elicited by the Parkinsonian neurotoxin, 1-methyl-4-phenyl-1,2,3,6-tetrahydropyridine (MPTP)

**DOI:** 10.1007/s00018-012-1071-9

**Published:** 2012-07-21

**Authors:** Kyoung-in Cho, Kelly Searle, Mason Webb, Haiqing Yi, Paulo A. Ferreira

**Affiliations:** 1 Department of Ophthalmology, Duke University Medical Center, DUEC 3802, 2351 Erwin Road, Durham, NC 27710 USA; 2 Department of Epidemiology, The Johns Hopkins Bloomberg School of Public Health, 615 N. Wolfe Street, Baltimore, 21205 MD; 3 Department of Pathology, Duke University Medical Center, Durham, NC 27710 USA

**Keywords:** Ran-binding protein 2 (RanBP2), MPTP neurotoxicity, Dopaminergic neurons, Gliosis, Metabolomics, Parkinson, Gene–environment interaction

## Abstract

Many components and pathways transducing multifaceted and deleterious effects of stress stimuli remain ill-defined. The Ran-binding protein 2 (RanBP2) interactome modulates the expression of a range of clinical and cell-context-dependent manifestations upon a variety of stressors. We examined the role of *Ranbp2* haploinsufficiency on cellular and metabolic manifestations linked to tyrosine-hydroxylase (TH^+^) dopaminergic neurons and glial cells of the brain and retina upon acute challenge to 1-methyl-4-phenyl-1,2,3,6-tetrahydropyridine (MPTP), a parkinsonian neurotoxin, which models facets of Parkinson disease. MPTP led to stronger akinetic parkinsonism and slower recovery in *Ranbp2* ^+/−^ than wild-type mice without viability changes of brain TH^+^-neurons of either genotype, with the exception of transient nuclear atypia via changes in chromatin condensation of *Ranbp2* ^+/−^ TH^+^-neurons. Conversely, the number of wild-type retinal TH^+^-amacrine neurons compared to *Ranbp2* ^+/−^ underwent milder declines without apoptosis followed by stronger recoveries without neurogenesis. These phenotypes were accompanied by a stronger rise of EdU^+^-proliferative cells and non-proliferative gliosis of GFAP^+^-Müller cells in wild-type than *Ranbp2* ^+/−^ that outlasted the MPTP-insult. Finally, MPTP-treated wild-type and *Ranbp2* ^+/−^ mice present distinct metabolic footprints in the brain or selective regions thereof, such as striatum, that are supportive of RanBP2-mediated regulation of interdependent metabolic pathways of lysine, cholesterol, free-fatty acids, or their β-oxidation. These studies demonstrate contrasting gene-environment phenodeviances and roles of *Ranbp2* between dopaminergic and glial cells of the brain and retina upon oxidative stress-elicited signaling and factors triggering a continuum of metabolic and cellular manifestations and proxies linked to oxidative stress, and chorioretinal and neurological disorders such as Parkinson.

**Electronic supplementary material:**

The online version of this article (doi:10.1007/s00018-012-1071-9) contains supplementary material, which is available to authorized users.

## Introduction

1-methyl-4-phenyl-1,2,3,6-tetrahydropyridine (MPTP) is a neurotoxin that models multiple facets of Parkinson’s disease (PD) [1–4]. 1-methyl-4-phenylpyridinium (MPP^+^) is the active metabolite of MPTP that is taken up by dopaminergic neurons [5], where it is thought to poison these by inhibition of the mitochondrial complex I and generation of superoxide anions [6, 7]. Previous experiments with mesencephalic neuronal cultures also supported that MPTP could promote the displacement of dopamine from vesicular stores, leading then to the intracellular and extracellular auto-oxidation of dopamine and production of reactive oxygen species (ROS) [8]. The pathophysiological relevance of these results are controversial, because (i) dopaminergic neurons (DA) of mice lacking functional Ndufs4, a subunit critical to the functional assembly of complex I, are not protected to MPP^+^ [9] and (ii) acute administration of MPTP still induces neurotoxicity in DA neurons of DA-deficient mice [10], whereas overexpression of the dopamine transporter in striatal neurons of transgenic mice lent support to the concept that impairment of intracellular dopamine vesicular sequestration suffices to promote neurodegeneration [11]. In addition to DA neuronal degeneration, MPTP is also reported to induce gliosis and microglial activation, events that are thought to contribute to the injury of DA neurons [12, 13]. The effects of MPTP and comparison of its pathophysiological outcomes become more complex with the observations that rodents are more resistant to MPTP neurotoxicity than primates and the multivariate susceptibilities to MPTP treatment vary significantly with mouse strains, sex, age, and treatment modalities [12, 14–20].

The neuroretina also harbors TH^+^-amacrine neurons. These DA neurons are the least numerous and the largest neurons in the retina because of the large arborization fields they present [21, 22]. Dopamine plays a role in light adaptation and PD patients suffer from various visual impairments [23, 24]. MPTP treatment is also reported to reduce reversibly or have no effect on the number of DA amacrine neurons and these effects are accompanied by a decrease of the retinal oscillatory potentials and activation of glial and microglial cells and astrocytes [25–30]. Hence, collectively these results support that the multifaceted and cell-context-dependent responses elicited by the Parkinsonian neurotoxin, MPTP, are susceptible to modulation by ill-characterized factors. Identification and characterization of such biological factors are of considerable therapeutic value, because among other reasons they may uncover disease-relevant pathways and serve as pharmacological targets to promote neuroprotection.

The Ran-binding protein 2 (RanBP2) is known to modulate directly responses elicited by various deleterious stressors, such as phototoxicity [31, 32], infectious agents [33], and carcinogens [34]. In this regard, haploinsufficiency of *Ranbp2* confers neuroprotection to photoreceptor neurons upon light-elicited oxidative stress [31, 32], whereas semi-dominant mutations in human *RANBP2* promote the rampant necrosis of basal ganglia neurons triggered by febrile states and viral infections of multiple etiologies [33]. Additionally, haploinsufficiency or hypomorphism of *Ranbp2* increases the susceptibility to tumorigenesis in the presence of carcinogens [34]. Parkin, whose loss-of-function causes PD in the human, is reported to associate with RanBP2 [35]. Like *Ranbp2*, *parkin* (*PARK2*) is also a tumor-suppressor gene [36]. Parkin and RanBP2 exhibit E3-ubiquitin ligase activity and the association of parkin with RanBP2 promotes the ubiquitination and degradation of RanBP2 [35, 37, 38]. Among other locations, RanBP2 and parkin appear to localize to the mitochondria, where parkin is thought to exert a neuroprotective role [39–42]. *parkin* ^−*/*−^ mice present deficits in lipid and glucose metabolism and energy homeostasis [42, 43], a phenotype that evokes manifestations observed with haploinsufficiency of *Ranbp2* [31, 40]. Hence, these data collectively support that RanBP2 modulates stress signaling pathways critical to neuronal viability and that RanBP2 and parkin may share pathomechanisms of relevance to the survival or function of dopaminergic neurons. To further define the role of deficits in RanBP2 in stress-mediated responses, we explored in this study the role of haploinsufficiency of *Ranbp2* in brain and retinal tyrosine-hydroxylase (TH^+^)-neurons and glial cells upon acute exposure to the Parkinsonian neurotoxin, MPTP, in an inbred mouse strain (129P2/OlaHsd). We found significant multifaceted phenotypic and metabolic footprint manifestations in the brain and retina between inbred wild-type and *Ranbp2* ^+*/*−^ mice. These phenotypic outcomes may underlie phenodeviances and pathobiological processes shared by diseases linked to oxidative stress, including parkinsonian manifestations.

## Materials and methods

### Mice and drug administration

Animal protocols were approved by the Institutional Animal Care and Use Committee at Duke University and the procedures adhered to the ARVO guidelines for the Use of Animals in Vision Research and National Academy of Sciences. Twenty-four to 28-week-old wild-type or *Ranbp2* ^+*/*−^ (*Ranbp2* ^+/*Gt(pGT0pfs)630Wcs*^) male mice previously generated with an identical inbred 129P2/OlaHsd genetic background were employed [40]. Mice were housed in a temperature-controlled and pathogen-free transgenic barrier facility in a standard 12:12 h light–dark cycle at <70 lux and given ad libitum access to water and breeder chow diet 5LJ5 (Purina). Mice received four bolus i.p. injections of MPTP^.^HCl (20 mg/kg, Sigma) in physiological saline at 2-h intervals and the first injection was administered 9:00–10:00 a.m. Mice were kept in isolation until being killed. For these studies, all mice were killed by cervical dislocation followed by decapitation at either 6 or 14 days after MPTP treatment.

### Open-field test

Mice were transferred separately to a 46 × 42 cm Plexiglas box with the bottom marked with a 25-square grid. Mice motility was recorded daily in isolation for 15 min; data were analyzed by counting the number of quadrants each mouse traveled within a constant time period.

### EdU administration, labeling, and staining

For cumulative EdU (5-ethynyl-2′-deoxyuridine, Invitrogen) labeling [44], mice received a daily bolus of EdU by i.p. injection (100 μl of 1 mg ml^−1^ EdU) during the first 6 or 13 days immediately following the last MPTP bolus or from days 9 to 13 after MPTP treatment. Mice were killed at days 6 or 14 after MPTP treatment. Development of EdU staining was performed always after incubation of sections or flatmounts with primary antibodies against cell markers and secondary antibodies. After incubation with the secondary antibody, brain and retinal sections or retinal flatmounts were fixed with 4 % paraformaldehyde in PBS solution for 15 min, washed once with 3 % BSA in 100 mM PBS, followed by permeabilization with 100 mM PBS, pH 7.4/0.5 % Triton X-100 for 20 min. Finally, specimens were incubated with the EdU detection cocktail for 30 min as per the manufacturer’s instructions (Click-iT^®^ EdU Alexa 488 Imaging Kit, Invitrogen, Carlsbad, CA, USA).

### Immunohistochemistry reagents

List of primary and secondary antibodies are described in Supplementary Materials and Methods.

### Tissue collection for morphological and morphometric analyses

Collection, processing, and immunostaining of brain and retina specimens for morphometric analyses are described in detail in Supplementary Materials and Methods.

### Immunohistochemistry

Free-floating 50-μm coronal sections of selected brain regions, 12-μm frozen retinal sections mounted on slides or flat-mount retinas were incubated in blocking buffer (PBS, pH 7.4, containing 0.1 % Triton X-100, 10 % normal goat serum) at room temperature for 1 h followed by an incubation with primary antibody in incubation buffer (PBS, pH 7.4, containing 0.1 % Triton X-100, 5 % normal goat serum) overnight at room temperature (brain) or 4 °C (retina). After three times of washing with washing buffer (PBS with 0.1 % Triton X-100) for 10 min, specimens were incubated in incubation buffer for 2 h with one or more Alexa-conjugated secondary antibody (1:1,000; Invitrogen, Carlsbad). Specimens were washed three times with washing buffer and whenever applicable mounted on glass slides for visualization and image acquisition with a Nikon 90i C1 Plus confocal microscope.

### TUNEL assays

Detection of apoptosis was carried out in situ in brain and retinal sections with the DeadEnd Fluorimetric TUNEL System (Promega) exactly as described elsewhere [31].

### Metabolomic profiling

Unbiased metabolomic profiling of whole brains of wild-type and *Ranbp2* ^+*/*−^ mice 6 days after MPTP treatment was performed by Metabolon, Inc. (Durham, NC). Brains were immediately stored at −80 °C after dissection and upon discarding the olfactory bulb and cerebellum. The brains were then provided to Metabolon, Inc. Additional metabolomic methodologies employed by Metabolon are described in Supplementary Materials and Methods.

### Biochemical assays

Immediately after the mice were killed, tissues were collected, snap frozen in liquid nitrogen, and stored at −80 °C in a freezer. Liver, midbrain, and striatum tissue extracts in 250 μl of NP-40 were prepared using Precellys^®^ 24 dual tissue homogenizer (Bertin Technologies, Montignyle-Bretonneux), at 6,000 rpm for 23 s. Total cholesterol, free cholesterol, and esterified cholesterol were measured as previously described [31]. Levels of Coenzyme A were determined by the Coenzyme A Assay Kit (Biovision, Mountain View, CA, USA) as directed by the manufacturer. Results were normalized against protein amounts in NP40 tissue extracts used for each assay. Two-sample *t* test statistical analyses with the assumption of unequal variance were performed; *p* ≤ 0.05 was defined as significant.

## Results

### Haploinsufficiency of *Ranbp2* causes increased akinesia and slow recovery upon MPTP treatment

**Fig. 1:**
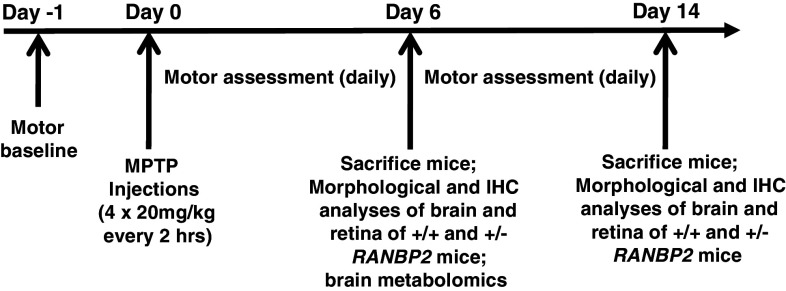
MPTP treatment and experimental time line. The baseline motor activities of wild-type and *Ranbp2* ^+*/*−^ mice were assessed the day prior to the MPTP treatment (*Day −1*). Wild-type and *Ranbp2* ^+*/*−^ mice on an inbred *129P2/OlaHsd* background underwent four bolus of MPTP i.p. injections spaced 2 h apart. The motor activities of the mice were assessed daily; brain and retinal tissues were collected for analyses at days 6 and 14 after MPTP treatment. Metabolic profiling was performed with brains of wild-type and *Ranbp2* ^+*/*−^ mice at day 6 after MPTP treatment

**Fig. 2:**
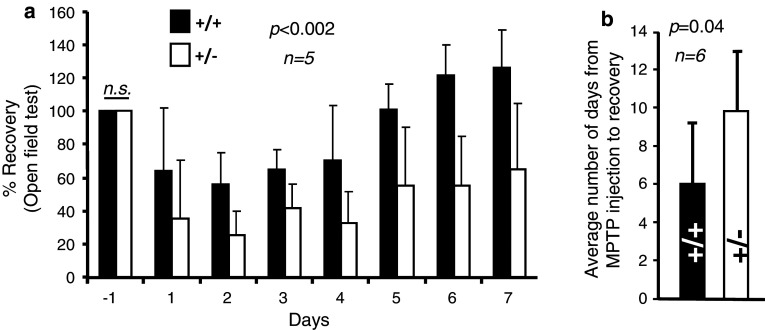
Motor activities of wild-type and *Ranbp2* ^+*/*−^ mice before and after MPTP treatment. **a** Motor activities between wild-type and *Ranbp2* ^+*/*−^ mice during the first 7 days after MPTP treatment. Wild-type and *Ranbp2* ^+*/*−^ mice exhibit no significant differences in basal motor activities a day prior to the initiation of MPTP treatment (*day* *−1*). The decline in motility reached its peak at the second day post-treatment and it plateaued for about 2 days before mice of both genotypes began to regain steadily their motilities. The greatest difference in motility between genotypes was observed at day 6 after MPTP treatment. **b** The recovery of the motility of wild-type mice is significantly faster than *Ranbp2* ^+*/*−^ mice. Data shown represent the mean ± SD; *p* < 0.002, *n* = 5 (**a**); *p* = 0.04, *n* = 6 (**b**). Black and white bars represent wild-type and *Ranbp2* ^+*/*−^ mice, respectively. *n.s.* not significant (*p* > 0.05); *+/+* wild-type, *+/−* *Ranbp2* ^+*/*−^

To assess the effects of MPTP on dopaminergic neurons and glial cells of the brain and retina of wild-type and haploinsufficient *Ranbp2* mice, 24-week-old inbred mice were subjected to a protocol of acute exposure of MPTP followed by time-course examination of their motility in an open-field test, state of TH^+^-neurons and glial cells of the brain and retina, and changes in brain metabolites (Fig. 1). As shown in Fig. 2a, there were no significant differences in basal locomotors activity between wild-type and *Ranbp2* ^+/−^ mice the day before MPTP treatment began, but there was a stronger decline followed by a slower recovery phase in *Ranbp2* ^+/−^ than wild-type mice after MPTP treatment. Further, not all *Ranbp2* ^+/−^ compared to wild-type mice recover their motility at the end of the 14-day experimental procedure (Fig. 2b).

### MPTP promotes transient nuclear atypia in brain TH^+^-neurons of *Ranbp2*^+/−^ mice

**Fig. 3:**
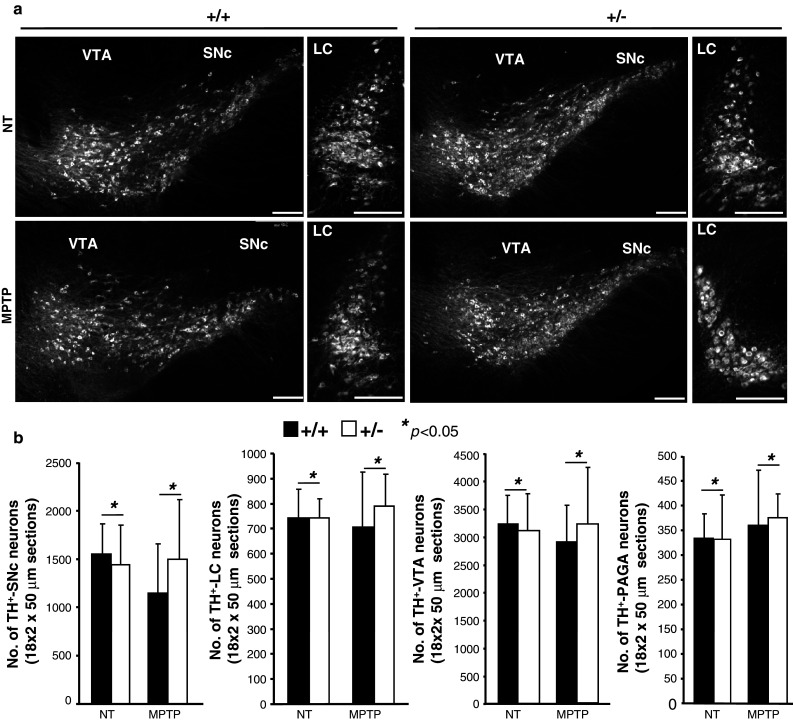
MPTP does not cause the loss of dopaminergic neurons in various regions of the brain in wild-type and *Ranbp2* ^+*/*−^ mice. **a** Confocal images of TH^+^-dopaminergic neurons of the substantia nigra pars compacta (*SNc*), ventral tegmental area (*VTA*), and locus coeruleus (*LC*). **b** Morphometric analyses show no differences in the number of cell bodies of dopaminergic neurons of the SNc, VTA, LC, and periaqueductal gray area (PAGA) between non-treated and MPTP-treated wild-type and *Ranbp2* ^+*/*−^ mice. Data shown represent the mean ± S.D, *p > 0.05, *n* = 5; MPTP-treated mice; *n* = 3, non-treated mice. MPTP-treated mice were analyzed 6 days after MPTP treatment. Cell body tallying reflects 18 coronal topographically equivalent sections of 50 μm from various regions of each brain hemisphere of wild-type and *Ranbp2* ^+*/*−^ mice. +/+ wild-type, +/−*Ranbp2* ^+*/*−^, *NT* non-treated; *scale bars* 150 μm

**Fig. 4:**
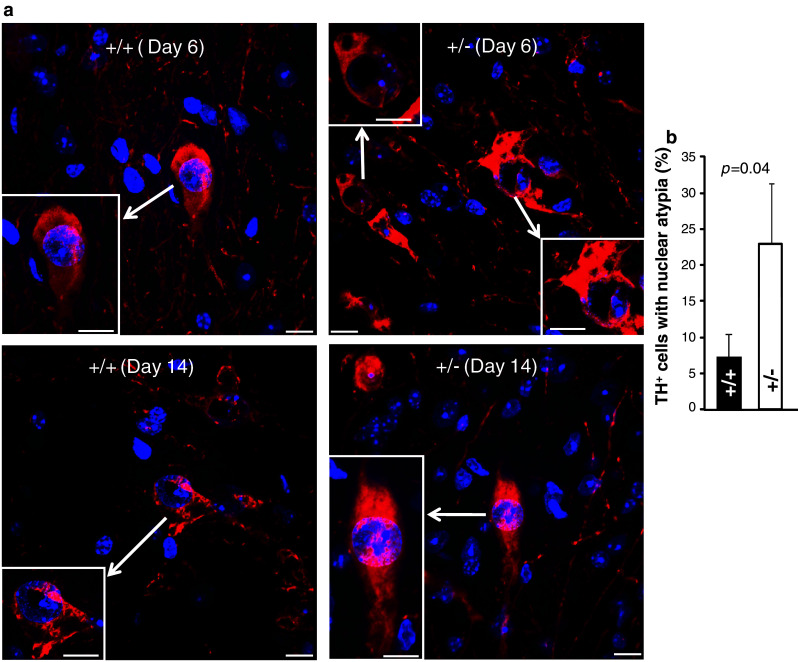
*Ranbp2* haploinsufficiency promotes transient nuclear atypia of TH^+^-dopaminergic neurons upon MPTP treatment. **a** Confocal images of SNc TH^+^-dopaminergic neurons (*red*) of wild-type and *Ranbp2* ^+*/*−^ mice counterstained with DAPI at 6 and 14 days after MPTP treatment. Note the asymmetric distribution of clumped chromatin and large nuclear areas voided of DAPI staining (nuclear vacuolization) in *Ranbp2* ^+*/*−^, but not wild-type mice, at 6-day post-MPTP treatment. These phenotypic differences between genotypes disappear at 14-day post-MPTP treatment. *Inset pictures* large magnifications of cell bodies of TH^+^-dopaminergic neurons denoted by *arrows*. *Scale bars* 10 μm. **b** Quantitative analysis of nuclear atypia of TH^+^-dopaminergic neurons between wild-type and *Ranbp2* ^+*/*−^ mice at 6-day post-MPTP treatment. Data shown represent the mean ± SD, *p* = 0.04, *n* = 3. +/+ wild-type, *+/−* *Ranbp2* ^+*/*−^

We then performed morphometric analyses of coronal sections between wild-type and *Ranbp2* ^+/−^ mice of cell bodies of TH^+^-neurons at the end of the 14-day experimental procedure and encompassing the following regions of the mesencephalic dopaminergic network: such as substantia nigra pars compacta (SNc), ventral tegmental area (VTA), locus coeruleus (LC), and periaqueductal gray area (PAGA) (Fig. 3a, b). We found no significant changes in the number of TH^+^-neurons between non-treated and treated mice of either genotype. We also examined the striatum, which contains axons and synaptic terminals of the TH^+^-neurons, for density changes of TH^+^-axons and -synaptic terminals. No remarkable differences in TH^+^-neurons were detected between treated mice of either genotype at 6 or 14 days after MPTP treatment (Supplemental Fig. 1). There was no evidence of apoptosis in all areas of the mesencephalic dopaminergic network by TUNEL assay (data not shown). Likewise, there was no up-regulation of glial fibrillary acidic protein (GFAP) throughout the brain sections examined, a process commonly elicited by neuronal injuries of multiple etiologies (data not shown) [45, 46]. The only distinct morphological difference was the development of nuclear atypia in TH^+^-neurons at 6 days after MPTP treatment that was significantly more pronounced in *Ranbp2* ^+*/*−^ than wild-type mice (Fig. 4a, b). The nuclear compartment became hypotrophic as reflected by the prominent and asymmetric clumping of DAPI-stained chromatin of TH^+^-neurons of *Ranbp2* ^+/−^ mice towards an edge of the nuclear compartment (Fig. 4a). These abnormalities disappear, however, by day 14 post-MPTP-treatment, since no differences in the nuclear distribution of DAPI-stained chromatin could be observed with mice of either genotype (Fig. 4a).

### MPTP promotes distinct effects between TH^+^-neurons and GFAP^+^-glia in retinae of wild-type and *Ranbp2*^+/−^ mice

**Fig. 5:**
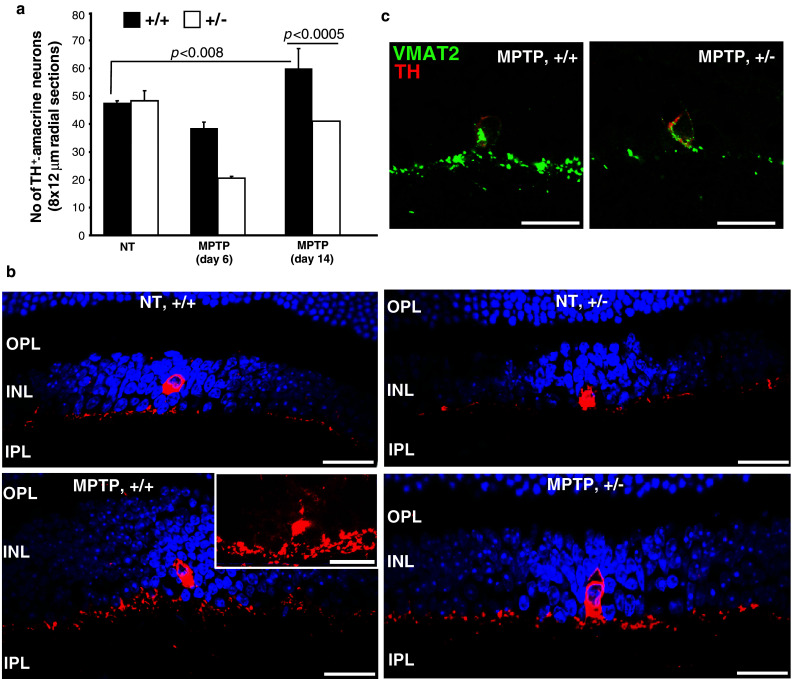
MPTP elicits a stronger decrease and a slower recovery of the number of retinal TH^+^-neurons in *Ranbp2* ^+*/*−^ than wild-type mice. **a** Comparison of the number of TH^+^-neurons in the retina between *Ranbp2* ^+*/*−^ and wild-type mice before and after MPTP treatment (6 and 14 days post-treatment). Note the stronger recovery of retinal TH^+^-neurons in wild-type mice 14 days after MPTP treatment surpasses the number of TH^+^-neurons observed in non-treated wild-type or *Ranbp2* ^+*/*−^ mice. Data shown represent the mean ± SD; *n* = 6, MPTP-treated mice; *n* = 3, non-treated mice. **b** Confocal images of retinal TH^+^-neurons (*red*) in non-treated (NT) and 14-day post-MPTP treated wild-type and *Ranbp2* ^+*/*−^ mice. Note the increase of the thickness of the TH^+^-synaptic plexus and the presence of weakly stained TH^+^-cell bodies surrounding a strongly stained TH^+^-amacrine cell body (*inset picture*) in wild-type mice 14 days after MPTP challenge. These weakly stained TH^+^-cell bodies were never observed in retinas of *Ranbp2* ^+*/*−^ mice. **c** The increase of the thickness of the TH^+^-synaptic plexus was accompanied also by a stronger increase of VMAT2 staining (*green*) in wild-type than *Ranbp2* ^+*/*−^ mice. +/+ wild-type, +/− *Ranbp2* ^+*/*−^; *scale bars* 25 μm

We also examined the effect of MPTP on TH^+^-amacrine neurons of the retina, since apparently conflicting reports exist on the reversible decrease of TH^+^-amacrine dopaminergic neurons upon MPTP treatment. No significant differences in the number of TH^+^-amacrine neurons exist between untreated wild-type and *Ranbp2* ^+/−^ mice (Fig. 5a). After 6 days of MPTP treatment, however, there were less TH^+^-amacrine neurons in both genotypes, but such a decrease was greater in *Ranbp2* ^+/−^ than wild-type mice (Fig. 5a). Such a decrease was not caused by the cell death of TH^+^-amacrine neurons, because we could not detect any TUNEL-positive cells in the inner nuclear or any other retinal nuclear layers (data not shown). Notably, the number of TH^+^-amacrine neurons of *Ranbp2* ^+/−^ recovered to levels comparable to those of non-treated mice by day 14 post-MPTP treatment, while in wild-type mice they exceeded by ~20 % those observed in non-treated mice (Fig. 5a). In association with this compensatory response, there was an increase of the thickness and intensity of synaptic plexus of TH^+^-amacrine neurons and the presence of weak immunoreactive TH^+^-neurons surrounding typically a strongly stained TH^+^-amacrine neuron in MPTP-treated wild-type, but not treated *Ranbp2* ^+/−^ and non-treated mice (Fig. 5b). It is noteworthy that these weak immunoreactive cells were never counted for the quantitative analyses described earlier. Likewise, these effects were accompanied by an increase in the staining of the dopaminergic synaptic plexus for the vesicular monoamine transporter-2 (VMAT2) (Fig. 5c). These changes were also highly selective toward TH^+^-amacrine neurons, because no significant changes were observed in calretinin immunostaining of AII amacrine cells and sublaminar synaptic layers of the inner plexiform layer and Cabp5 immunostaining of rod and cone bipolar neurons in the inner nuclear layer (data not shown).

**Fig. 6:**
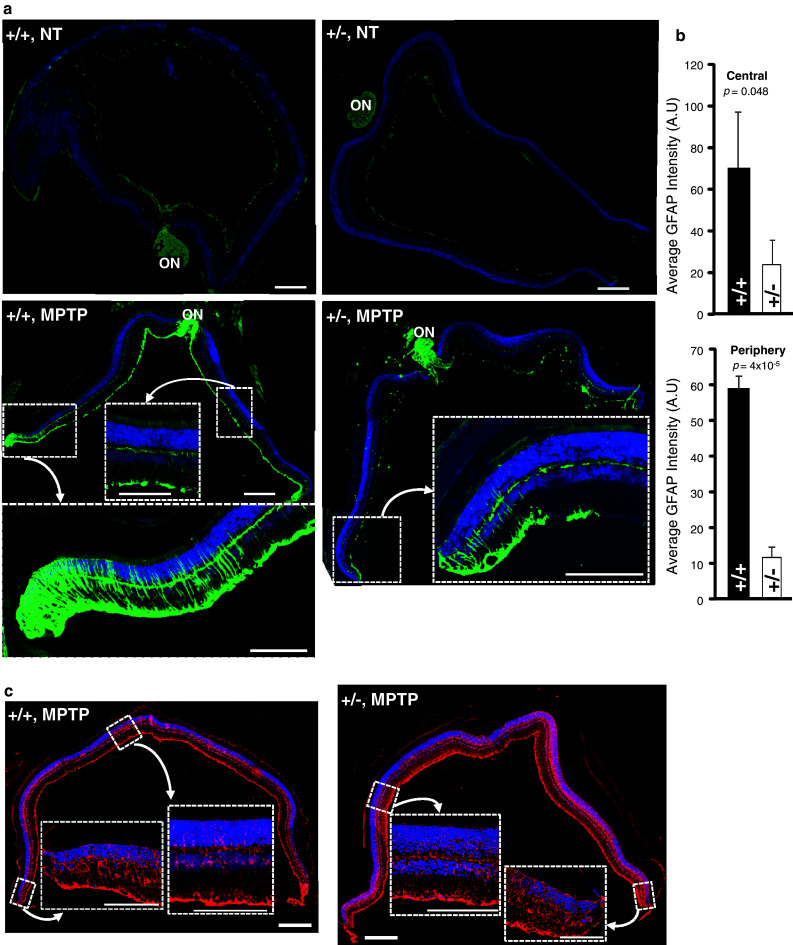
*Ranbp2* haploinsufficiency suppresses MPTP-elicited gliosis in the retina. **a** Non-treated mice exhibit extremely weak GFAP staining of Müller cells across the retina. In contrast, there is a prominent centrifugal increase of the number of GFAP^+^-Müller cells in retinas of MPTP-treated wild-type mice that are strongly decreased in *Ranbp2* ^+*/*−^ mice 14 days after MPTP treatment. Strong GFAP immunoreactivity is also observed in the optic nerve head (ON). *Inset pictures* denoted by the *arrows* are high magnifications of boxed peripheral or central regions of the retina. **b** Quantitative analyses of immunofluorescence intensity of GFAP^+^-Müller cells in central and peripheral regions of retinas of wild-type and *Ranbp2* ^+*/*−^ mice 14 days after MPTP treatment (as shown in **a**). Data shown represent the mean ± SD; *n* = 3. **c** The significant differences in the density of GFAP^+^-Müller cells observed between wild-type mice and *Ranbp2* ^+*/*−^ mice in **a** are not accompanied by differences in staining of the canonical glial (Müller) marker, glutamine synthetase, which, in contrast to GFAP, stains uniformly the glial cells across the retina. *Inset pictures* denoted by the *arrows* are high magnifications of peripheral or central regions of the retina. *+/+* wild-type, +/−*Ranbp2* ^+*/*−^, *A.U.* arbitrary units, *ON* optic nerve head; *scale bars* 200 μm

Stress stimuli of multiple etiologies and causing retinal damage are known to induce the up-regulation of glial fibrillary acidic protein (GFAP) in Müller glial cells [46]. Further, these glial cells are thought to present proliferative capacity and few appear to regenerate into amacrine neurons [46]. Hence, we examined whether MPTP treatment promoted changes in the density of GFAP^+^-radial glia (Müller cells) of the retina and these cells were the source of the increase in the number TH^+^-amacrine neurons in MPTP-treated wild-type mice. No remarkable changes of GFAP^+^-Müller cells were seen before and at day 6 after treatment in wild-type and *Ranbp2* ^+/−^ mice compared to non-treated mice (Fig. 6a, upper panel; data not shown). However, there was a robust and significant rise of GFAP^+^-glial cells in retinas of wild-type, but not *Ranbp2* ^+/−^ mice, at day 14 after MPTP-treatment (Fig. 6a) that was most prominent and significant toward the marginal regions of the retinas (Fig. 6a, lower panel; b), even though Müller cells immunostained for its canonical glial marker, glutamine synthetase, were distributed evenly throughout the retina regardless of the genotype (Fig. 6c). By contrast, the rise of GFAP^+^-Müller cells in treated *Ranbp2* ^+/−^ mice is very tenuous, with only a few GFAP^+^-Müller cells observed at the very marginal and optic nerve head regions of the retina (Fig. 6a, b). Hence, RanBP2 insufficiency suppresses gliosis upon MPTP challenge and this effect outlasts the neurotoxic insult without affecting the general distribution and viability of Müller glial cells.

### Haploinsufficiency of *Ranbp2* suppresses the proliferation of EdU^+^-cells in the retina that do not regenerate into TH^+^-amacrine neurons upon MPTP treatment

**Fig. 7:**
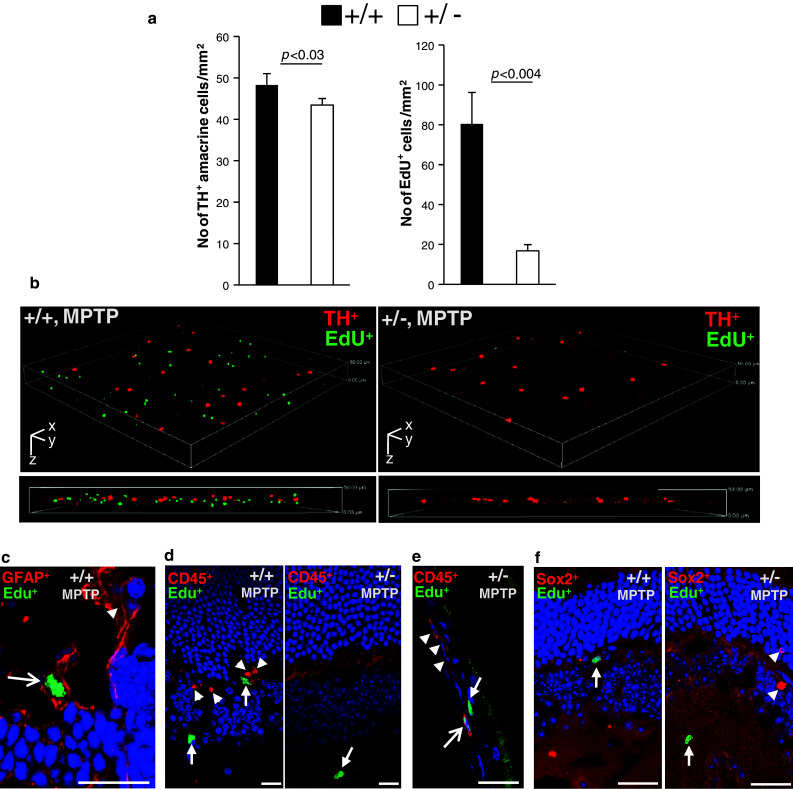
Retinal cellular proliferation elicited by MPTP is suppressed by insufficiency of RanBP2 and it lacks canonical dopaminergic, inflammatory, and vascular cellular markers. **a** The decrease of the number of TH^+^-amacrine neurons is accompanied by a strong decrease of EdU^+^-proliferating cells in *Ranbp2* ^+*/*−^ compared to wild-type mice 6 days after MPTP treatment. Data shown was collected from images of flat-mount retinas and represent the mean ± SD; *n* = 4, wild-type; *n* = 5, *Ranbp2* ^+*/*−^. **b** 3-D reconstruction of confocal *x*-*y*-*z* image stacks across a 50-μm depth of the inner nuclear and plexiform layers of the retina and depicting the non-overlapping spatial arrangement of cell bodies of TH^+^-amacrine neurons (*red*) and EdU^+^-cells (*green*) 6 days after MPTP treatment in wild-type and *Ranbp2* ^+*/*−^ mice. *Ranbp2* ^+*/*−^ mice present far fewer EdU^+^-cells than do wild-type mice.* The images below the 3-D images* are 2-D cross sections of collapsed 3-D images. *Scale bars*: *x* 635.5 μm, *y* 635.5 μm, *z* 50 μm with cell bodies of TH^+^-amacrine neurons occupying the mid-plane of the *z*-stack. **c** Only few GFAP^+^-cells (*red*) in the retina were co-labeled by EdU (*green*) in wild-type mice; GFAP^+^-cells are shown with (*arrow*) and without (*arrowhead*) EdU labeling. **d** No CD45^+^-cells (*red*, *arrowhead*) in the retina were observed to be labeled by EdU (*green*, *arrow*) in mice of either genotype, although in the choroid plexus few CD45^+^-cells were co-labeled by EdU (*arrow*) (**e**). **f** No Sox2^+^-cells (*red*, *arrowhead*) in the retina were observed to be labeled by EdU (*green*, *arrow*) in mice of either genotype. Images **c**–**f** are representative retinal sections of mice provided with a daily EdU bolus for 13 days after MPTP treatment. +/+ wild-type, +/− *Ranbp2* ^+*/*−^

The suppression of the recovery phase of TH^+^-amacrine neurons in *Ranbp2* ^+/−^ mice at days 6 and 14 after MPTP treatment (Fig. 5a) hints that insufficiency of RanBP2 suppresses the proliferation of progenitor retinal cells capable of regenerating into TH^+^-amacrine neurons or the differentiation of existing cells by the up-regulation of dopaminergic markers. Hence, we analyzed retinas of mice that underwent MPTP treatment and three different regimens of injections with the thymidine analog, 5-ethynyl-2-deoxyuridine (EdU), which incorporates into the DNA of proliferative cells [44]. The EdU modality treatments comprised daily EdU injections during the first 5 days, between days 9 and 13, or throughout the 13 days of the experimental procedure. Retinas were then analyzed the day after the last EdU injection. Quantitative analyses of confocal images scanned across a 50-μm depth of the mid-plane of flatmount retinas of 5-day EdU-treated mice showed that *Ranbp2* ^+/−^ had a significantly less number of EdU^+^-cells than wild-type mice (Fig. 7a). Such a genotype-dependent decrease of proliferating cells was also qualitatively observed with the other two regimens of EdU-treatment. Regardless of the EdU-regimen modality and genotype, we never detected co-labeling of single TH^+^-amacrine neurons with EdU (Fig. 7b). These data support that the changes in the number of TH^+^-amacrine neurons result from the genotype-dependent modulation of expression of TH, and possibly of other dopaminergic markers, such as VMAT2, rather than from the proliferation of progenitor cells.

We then examined the identity of the EdU^+^- cells, whose proliferation was suppressed in *Ranbp2* ^+/−^ mice upon MPTP treatment, with antibodies against antigens known to be either expressed in retinal progenitor cells or markers up-regulated in proliferating cells upon retinal injury. In particular, Sox2 is known to be required for differentiation of progenitor cells to amacrine and other retinal neurons [47], whereas GFAP, CD45, and CD11b are markers for Müller glia and astrocytes, vascular (leukocytes), and resting and activated microglia and macrophages, respectively [48–50]. GFAP^+^-, CD45^+^-, and CD11b^+^-cells are known to proliferate upon retinal damage to various stress stimuli [50–52]. As shown in Fig. 7c–f, most cellular markers tested were expressed in EdU^−^ cells, albeit with some differences. Among the GFAP^+^ cells, only very few were EdU^+^, thus supporting GFAP^+^-cells represent a small fraction of EdU^+^-proliferating cells (Fig. 7c). In the retina, all CD45^+^ were EdU^−^ (Fig. 7d) and the few CD45^+^EdU^+^ identified in several sections were exclusively localized in the choroid plexus (Fig. 7e). All CD11b^+^ cells identified were EdU^−^cells and they appeared to be macrophages that were also exclusively localized in the choroid plexus (Supplementary Fig. 2). Finally, we did not identify EdU^+^-Sox2 cells (Fig. 7f). Hence, the majority of the EdU^+^-cells in the retina represents cells of novel origin or identity.

### *Ranbp2*-dependent metabolic imbalances in the brain upon exposure to acute MPTP treatment

**Fig. 8:**
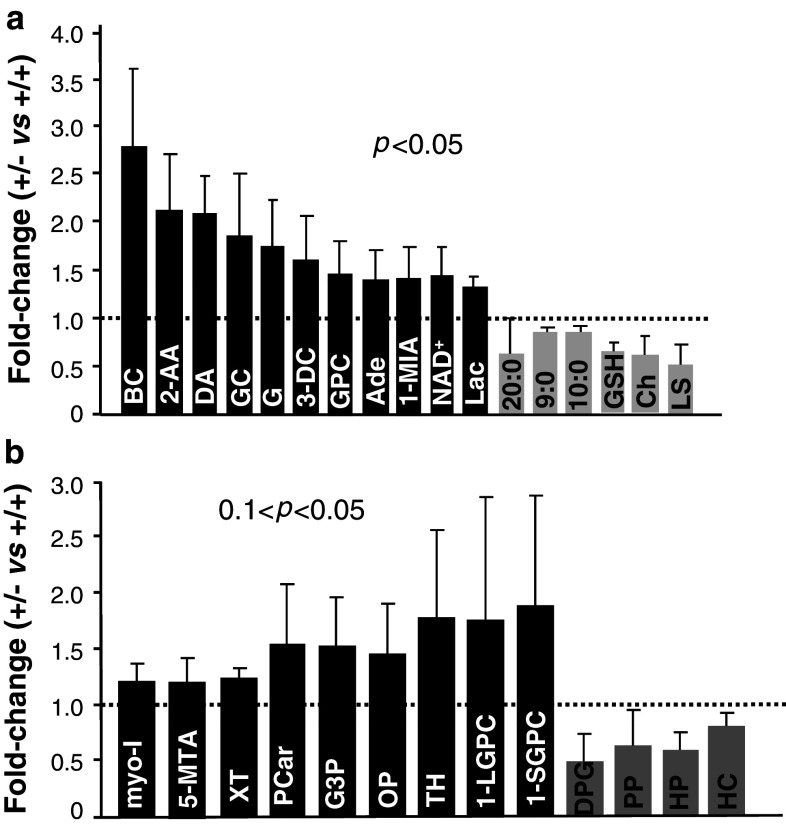
Metabolomic alterations between *Ranbp2* ^+*/*−^ and wild-type mice upon MPTP treatment. **a** Fold-changes in brain metabolites of *Ranbp2* ^+*/*−^ relative to wild-type mice 6 days after the acute MPTP insult. Data represent the mean ± SD and metabolites with significant variations (Wilcoxon *p* < 0.05), *n* = 5 for all metabolites but BC, DA, Lac, GSH, and Ch (*n* = 5, wild-type; *n* = 4, *Ranbp2* ^+*/*−^), G (*n* = 4, wild-type; *n* = 5, *Ranbp2* ^+*/*−^) and NAD^+^ (*n* = 4). **b** Fold-changes in brain metabolites of *Ranbp2* ^+*/*−^ relative to wild-type mice 6 days after MPTP challenge that narrowly missed or approached statistical significance (Wilcoxon 0.1 < *p* < 0.05). Data represent the mean ± SD, *n* = 5. *Black* and *grey bars* represent higher and lower levels of metabolites in *Ranbp2* ^+*/*−^ relative to wild-type mice, respectively. *GPC* glycerophosphorylcholine, *1-MIA* 1-methylimidazoleacetate, *3-DC* 3-dehydrocarnitine, *GC* glutaroyl carnitine, *2-AA* 2-aminoadipate, *20:0* arachidate, *9:0* pelargonate, *10:0* caprate, *LS* lathosterol, *Ade* adenine, *DA* dehydroascorbate, *BC* butyrylcarnitine, *G* glucose, *L* lactate, *NAD* ^*+*^ nicotinamide adenine nucleotide, *GSH* glutathione (reduced), *Ch* cholesterol, *1-LGPC* 1-linoleoylglycerophosphocholine, *1-SGPC* 1-stearoylglycerophosphocholine, *DPG* 1,3-dipalmitoylglycerol, *PP* phosphopantetheine, *HP* hippurate, *HC* homocarnosine, *TH* threonate, *myo-I* myo-inositol, *5-MTA* 5-methylthioadenosine, *Pcar* propionylcarnitine, *G3P* glycerol 3-phosphate, *XT* xanthosine, *Lac* lactate, *OP* ophthalmate

In light of the temporal differences in behavioral and cellular phenotypes observed between inbred wild-type and *Ranbp2* ^+/−^ mice upon MPTP treatment, we took an unbiased metabolomic approach to examine differences in metabolite profiles that contribute to genotype-dependent manifestations outlasting the effects of MPTP. We employed brain tissue without the olfactory bulb and cerebellum, because of the amount of tissue needed to perform metabolomic analysis, a requirement the retinal tissue does not meet, and no differences in the gross cellular composition of the brain were observed between mice of either genotype, an outcome that would otherwise skew the metabolic profile. We performed metabolomics on mice 6 days after MPTP treatment, because this was the time point when there was the greatest difference in locomotor activity between wild-type and *Ranbp2* ^+/−^ mice (Fig. 2a). This metabolomic approach led to the identification of 17 metabolites, which were significantly altered between treated wild-type and *Ranbp2* ^+/−^ mice with an identical inbred 129P2/OlaHsd genetic background (*p* < 0.05) (Fig. 8a), and of another 13 metabolites, which narrowly missed the *p* < 0.05 cutoff (e.g., hippurate, myo-inositol, homocarnosine, phosphopantetheine with *p* = 0.0535, 0.0538, 0.0618, and 0.066, respectively) or with trends approaching significance (0.05 < *p* < 0.10) (Fig. 8b). The inclusion of trend differences further aided the interpretation of changes in metabolites and metabolic pathways deemed to undergo significant changes.

**Fig. 9:**
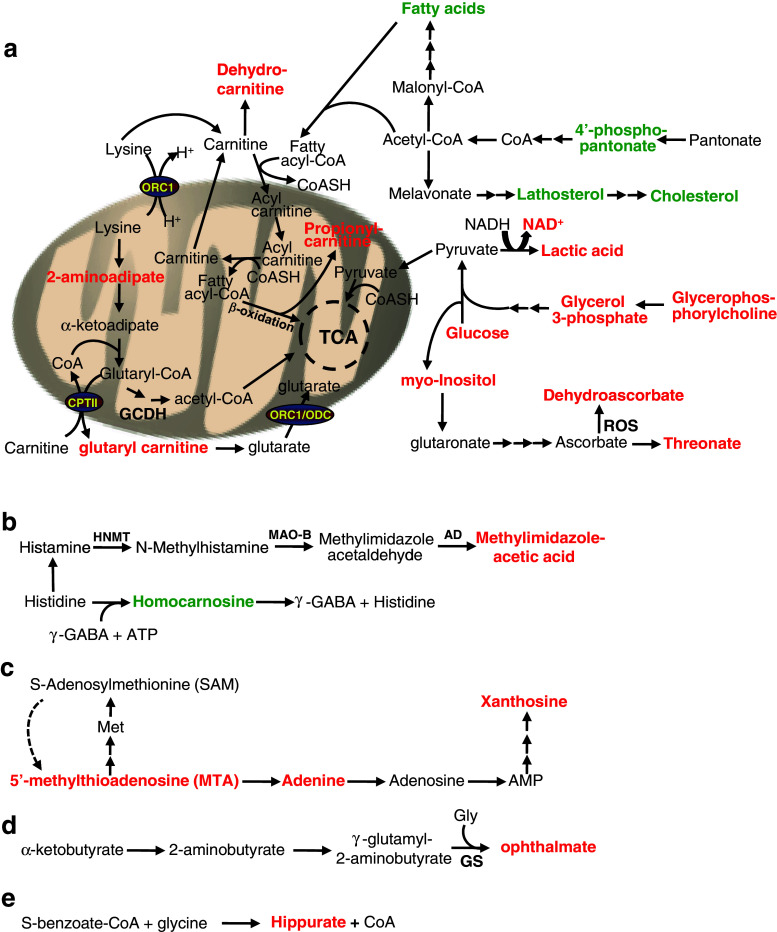
Model of metabolic deregulation in the brain by RanBP2 insufficiency upon MPTP insult. Alterations of brain metabolites by RanBP2 insufficiency upon acute MPTP exposure were subdivided into five major groups (**a–e**). Metabolites of group **a** participate in depicted multistep and interdependent metabolic pathways regulating bioenergetics in the cytosol or mitochondria related to lipid and glucose metabolism and cofactors thereof. These metabolites can be further divided into three subsets: lipid metabolites or precursors and cofactors thereof, acylcarnitine conjugates and metabolites of lysine catabolism, bioenergetic metabolites/substrates, and cofactors thereof (e.g., glycerophosphorylcholine, glucose, lactate, NAD^+^) that likely promote reduced anaplerosis of the Krebs cycle. Metabolites of group **b** reflect changes in histidine catabolic pathways, whereas those of group **c** represent variations in the methionine and adenine salvage pathways. Metabolites of groups **d** and **e** represent changes in conjugation reactions with glycine, reflecting variations of redox pathways (group **d**) and detoxification reactions (group **e**). See text for further details. Metabolites in *red* and *green* are, respectively, up-regulated and down-regulated; *single* and *multiple arrows* represent single and multi-step pathways. *MAO-B* monoamine oxidase, *type B* *ORC1* ornithine carrier, *CPTII* carnitine palmitoyltransferase II, *ODC* oxodicarboxylate carrier, *OGC* oxoglutarate carrier

The variations in metabolites observed between genotypes upon MPTP treatment could be divided into five major groups based on the role(s) and mapping of a set of metabolites into metabolic pathways according to the Kyoto Encyclopedia of Genes and Genomes (KEGG) (Fig. 9). The first group comprises three metabolite subsets, whose levels are thought to affect interdependent metabolic pathways. The first subgroup is characterized by a decrease of lipid metabolites or precursors thereof (Figs. 8, 9a). In particular, we found a significant decrease of two essential medium-chain free fatty acids, pelargonate (9:0) and caprate (10:0) (0.85-fold), and the long-chain free fatty acid, arachidate (20:0) (0.75-fold) in *Ranbp2* ^+/−^ mice. Likewise, there is a strong and significant decrease in lathosterol (0.53-fold), a canonical whole-body indicator of cholesterol synthesis [53, 54]. Indeed, a significant decrease of cholesterol levels (0.61-fold) was observed. These deficits were also accompanied by a trend for a decrease of the levels of phosphopantetheine (0.63-fold), a key precursor for the synthesis of the cofactor, coenzyme (CoA), which is critical for the synthesis of free fatty acids and cholesterol [55]. It is of interest to note that the fold-changes in levels of CoA and one of its intermediate precursors, 3′-dephosphocoenzyme A, had the same magnitude of phosphopantetheine (0.63-fold), even though the differences of the mean levels of 3′-dephosphocoenzyme A and CoA between genotypes were not statistically significant.

The second subset comprises a significant rise in *Ranbp2* ^+/−^ mice of the levels of several carnitine conjugates, such as 3-dehydrocarnitine (1.6-fold) and glutaroyl carnitine (1.87-fold), and a trend for an increase in propionylcarnitine (1.55-fold). These surges are also accompanied by an increase of 2-aminoadipate (2.13-fold), an intermediate in lysine metabolism. The rise of the metabolites of this group is likely due to an increase of lysine catabolism, because of increases in the levels of 2-aminoadipate, 3-dehydrocarnitine, and glutaroyl carnitine. Further, odd-chain acylcarnitines, such as propionylcarnitine (C3), are produced during amino acid catabolism [56]. Interestingly, we found the levels of several other acylcarnitines, such as butyrylcarnitine, acetylcarnitine, palmitoylcarnitine, stearoylcarnitine, and oleoylcarnitine, to be elevated in *Ranbp2* ^+/−^ mice. The changes in these acylcarnitines levels represented some of the greatest fold increases of this study (from 1.55- to over threefold) and there was a significant correlation between their elevated levels in *Ranbp2* ^+/−^ mice (*r*_s_ > 0.9; *p* < 0.037) even though the differences of the mean levels of each between genotypes were not significant. Acylcarnitines transport long-chain and medium-chain fatty acids into the mitochondria for energy generation via β-oxidation and together with reduced levels of coenzyme A, they likely contribute to the accumulation of acylcarnitines [57].

Finally, the last subgroup is characterized by significant increases of glycerophosphorylcholine (1.48-fold), glucose (1.76-fold), lactate (1.36-fold), nicotinamide adenine dinucleotide (NAD^+^) (1.45-fold), and dehydroascorbate (2.1-fold). These changes are accompanied also by increased trends of the levels of glycerol 3-phosphate (1.54-fold), myo-inositol (1.22-fold), and threonate (1.78-fold). Altogether, the accumulation of these metabolites may arise from an impairment of the glycerophosphate shunt and reduced anaplerosis of the Krebs cycle [58]. Glycerophosphorylcholine and myo-inositol are also cellular osmolytes with important roles in maintaining cellular volume in response to hypertonic stress [59].

The rise in lysine catabolism in *Ranbp2* ^+/−^ mice also correlates directly with changes in a second group of metabolites, 1-methylimidazoleacetate (t-MIAA; 1.43-fold) and homocarnosine/γ-aminobutyryl-l-histidine (0.81-fold) (Figs. 8, 9b). The significant increase of the former results from the enhanced catabolism of histidine to histamine and it is a reliable marker of histamine turnover rate [60] and such augmented conversion likely induces a decrease of the levels of the dipeptide homocarnosine [61]. This dipeptide is formed from γ-aminobutyric acid (GABA) and histidine, and it is found mostly in the brain, where it may act as a GABA reservoir, the main neurotransmitter inhibitor of the brain. Notably, the immediate precursor of t-MIAA, methylimidazole acetaldehyde, is the metabolite of the oxidative deamination of *N*-methylhistamine by the monoamine oxidase type B (MAO-B), which also metabolizes dopamine upon its oxidative deamination and converts MPTP to MPP^+^. MAO-B is also a therapeutic target for Parkinson’s disease.

The third group reflects a rise of metabolites of the methionine and adenine salvage pathways in *Ranbp2* ^+/−^ mice (Figs. 8, 9c). There was a significant 1.4-fold increase of adenine that was accompanied by strong trends of a rise of the levels of 5-methylthioadenosine (1.32-fold) and xanthosine (1.32-fold). Finally, the fourth and fifth groups are represented by the metabolites, reduced glutathione (GSH) (0.65-fold) and ophthalmate (1.47-fold), and hippurate (0.59-fold), respectively (Figs. 8, 9d, e). Ophthalmate and hippurate metabolites arise from the conjugation reactions with glycine. Hippurate is a common metabolite of detoxification reactions [62]. Ophthalmate is a tripeptide analogue of glutathione and a useful biomarker of redox state and oxidative damage of tissues when it is accompanied typically by depletion of reduced glutathione (GSH) [63]. We found that lower levels of GSH were accompanied by a trend of higher levels of ophthalmate. Further, it is of interest to note that the glutathione cycle is also coupled with the GABA shunt (synthesis of γ-aminobutyrate) [64], thus changes in the levels of metabolites of glutathione cycle are likely to contribute to compound biochemical phenotypes.

**Fig. 10:**
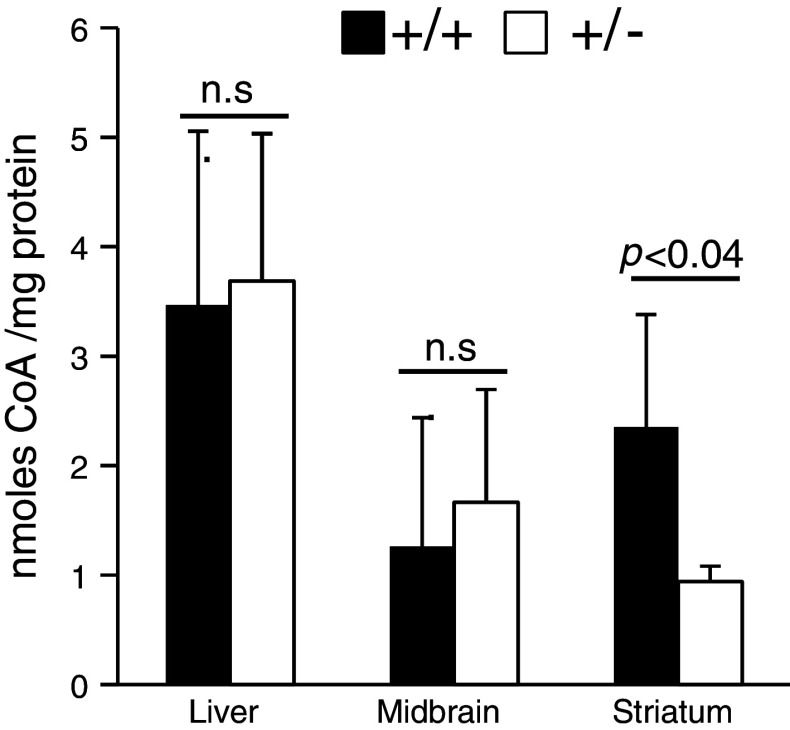
Haploinsufficiency of *Ranbp2* selectively causes a decrease of the levels of CoA in the striatum upon MPTP insult. CoA levels were decreased selectively in the striatum of *Ranbp2* ^+*/*−^ mice relative to wild-type mice 6 days after the acute MPTP insult. No changes of CoA levels were observed in the midbrain and liver. Data shown represent the mean ± SD; *n* = 4, wild-type; *n* = 5, *Ranbp2* ^+*/*−^. Black and white bars represent wild-type (*+/+*) and *Ranbp2* ^+*/*−^ (+/−) mice, respectively

The depressed levels of selective free fatty acids species and the accumulation of several acylcarnitine species in the brain of *Ranbp2* ^+/−^ mice may result in part from changes in the limited free pool of CoA or intermediates thereof in the cytosol and mitochondria. Hence, we examined whether there were tissue differences in deficits of CoA in the liver, the major organ of CoA synthesis, the striatum, where the uptake and accumulation of MPP^+^ takes place, and the midbrain, where a large number of dopaminergic cell bodies (e.g., substantia nigra) are clustered. We found the levels of CoA to be decreased over twofold in the striatum of *Ranbp2* ^+/−^ mice, but not changed in the midbrain and liver (Fig. 10).

## Discussion

The data support that a difference in genetic dosage of *Ranbp2* in the inbred *129P2/OlaHsd* genetic background modulates significant metabolic pathways in the brain without causing significant nigrostriatal cellular changes upon acute MPTP exposure. In contrast, a variety of cell types of the retina undergo significant cellular changes upon *Ranbp2* haploinsufficiency and MPTP exposure with the most striking effects observed by the suppression of the following cellular manifestations: (i) gliosis of GFAP^+^-Müller cells outlasting the xenobiotic insult, (ii) recovery of TH^+^-amacrine neurons and (iii) activation of a heterogeneous population of proliferative cells across the retina. This study also uncovers a distinct and large proliferative population of cells elicited by MPTP that differ from those reported for other neurotoxicity models, such as intraocular injection of *N*-methyl-D-aspartic acid (NMDA) with or without selective growth factors, glutamate, or α-aminoadipate [65–67]. NMDA together with retinoic acid induces the regeneration of a restricted number of Müller cells into bipolar or photoreceptor cells, whereas NMDA in the presence of EGF, FGF1, or FGF1 combined with insulin, stimulates the production of Müller glia with a small fraction of these regenerating into undefined amacrine neurons [65, 66]. However, glutamate or α-aminoadipate, which was found in this study to be significantly elevated in *Ranbp2* ^+*/*−^, promotes the transient proliferation of Müller glial cells and differentiation into retinal neurons, such as photoreceptors [67]. By contrast, our study found that MPTP elicits long-lasting retinal gliosis by dramatically up-regulating resident GFAP^+^-Müller cells without the majority of these cells undergoing proliferation. Further, we did not identify any MPTP-elicited EdU^+^-proliferating cells with the capacity to regenerate into TH^+^-amacrine neurons even though most were localized to the inner retina. Instead, MPTP promotes the transient suppression of TH expression in dopaminergic amacrine neurons followed by marked and compensatory recoveries of TH expression in such neurons, a finding that is consistent with the work reported by Tatton et al. [25]. Hence, the data imply the existence of distinct cellular and molecular responses to MPTP-neurotoxicity between TH^+^-neurons of the brain and the retina. These data support the existence of distinct TH^+^-neuronal subtypes between these tissues and RanBP2- and microenvironment-dependent trophic signaling between unique populations of MPTP-activated and proliferative glia in the brain and retina.

These data also support the existence of a novel population of EdU^+^-proliferating cells elicited by MPTP and repressed by *Ranbp2* haploinsufficiency. In contrast to other inflammatory models of neurotoxicity and injury to the retina [50–52, 68], the EdU^+^-proliferating cells elicited by MPTP and resident in the retina were neither CD45^+^ nor CD11b^+^ and the former cells were never detected in retinas of *Ranbp2* ^+*/*−^ mice. Instead, CD45^+^EdU^−^ and CD45^+^EdU^+^ cells were only detected in the choroid (vascular) plexus of *Ranbp2* ^+*/*−^ mice, whereas CD11b^+^ cells were exclusively present in the choroid plexus and they were all EdU^−^. Altogether, these data strengthen the notion of the development of distinct and antagonistic phenotypic outcomes observed between cell types by Ranbp2 insufficiency upon other disease stressors, such as phototoxicity [31, 32] and carcinogen exposure [34]. For example, the increased carcinogen-induced cellular proliferation (e.g., tumorigenesis) upon Ranbp2 insufficiency contrasts sharply with the suppression of the MPTP-induced proliferative capacity of progenitor cells in the retina of *Ranbp2* ^+*/*−^ mice as uncovered by this study. These differences are likely caused by the deregulation of the Ranbp2-mediated nucleocytoplasmic shuttling of factors or receptors thereof controlling cell proliferation or differentiation [69–73]. One of such factors is the Ranbp2 partner, Ubc9, which is an E2-ligase implicated in the shuttling and homeostasis of some orphan receptors [32, 74–78]. It is noteworthy that Ubc9 mediates the repression of pro-inflammatory responses in microglia and astrocytes by Nurr1 [79], which is associated with familial late-onset PD [80].

Our studies also reveal metabolic imbalances in the brain caused by haploinsufficiency of *Ranbp2* upon acute exposure to MPTP. The metabolic footprint uncovered is significant, because it not only validates and opens new venues to the identity of some undefined and possibly shared metabolites modulated by other stressors, such as phototoxicity [31, 32], but it also delineates disease and diagnostic predictors of metabolic pathways with relevance to the understanding of complex clinical and subclinical phenotypes of human diseases caused by interactions between the environment and *Ranbp2*.

In this regard, this study defines the identity of two medium-chains (pelargonate and caprate) and a long-chain fatty acid species (arachidate/eicosanoic acid), whose decreased levels are associated with haploinsufficiency of *Ranbp2*. Interestingly, the level of arachidate increases upon inflammation [81, 82] and as such, its decrease in *Ranbp2* ^+*/*−^ upon MPTP treatment may contribute to the suppression of inflammatory cellular responses observed in the retina of *Ranbp2* ^+*/*−^ mice. Further, the modulation of pro-inflammatory responses linked to insufficiency of RanBP2 is likely mediated by the concerted action of multiple metabolites. 5-methylthioadenosine and α-aminoadipate, which are increased in *Ranbp2* ^+*/*−^ mice, are additional metabolites with important anti-inflammatory or anti-proliferative properties, or both [67, 83, 84].

This work also identifies a set of metabolites from metabolic pathways, which are apparently distinct, and are known to either produce biomarkers of oxidative stress or present secondary and protective properties against toxic or injury insults of other etiologies. For example, l-propionyl carnitine is associated with a reduction in lipid hydroperoxides and superoxide anions and the propionyl moiety of l-propionyl carnitine provides intermediates to the TCA cycle upon stress conditions and contributes to its therapeutic benefits [57]. Homocarnosine and 5-methylthioadenosine are also known to be protective against oxidative stress with the former acting also as a scavenger for reactive oxygen species [61, 83]. The increase of these metabolites is accompanied by disruption of redox homeostasis as reflected by an increase of diagnostic markers of oxidative stress, such as dehydroascorbate and ophthalmate, with the latter acting as biomarker of glutathione depletion [63]. Our data support that the rise of such “protective” metabolites likely represents secondary and compensatory responses and diagnostic markers to noxious insults and that insufficiency of RanBP2 confers an increased susceptibility to such neurotoxic damage in the brain.

Finally, the accumulation of various acyl-carnitines, glucose, lactic acid, and NAD^+^ support a detoxifying efflux from the mitochondria of metabolites derived from the incomplete β-oxidation of free fatty acids concomitant with enhanced anaerobic glycolysis. Current knowledge supports that these imbalances arise likely from the glia, because neurons are thought not to utilize β-oxidation of free fatty acids as energy source [85]. These bioenergetic manifestations assume heightened relevance to our studies, because MPP^+^ is thought to inhibit directly the NADH:ubiquinone oxidoreductase of Complex I of the electron transport chain of the mitochondria [6, 7] and the decrease of the levels of CoA observed selectively in the striatum of *Ranbp2* ^+*/*−^ mice may contribute to the incomplete β-oxidation of free fatty acids in this brain region. It is not exactly clear how partial deficits of RanBP2 exacerbate certain bioenergetic facets of neurotoxic stress-induced manifestations. However, the work of Neilson et al. and ours support that the down-regulation of hexokinase I activity by deficits of RanBP2 [40] or stimulation of the uncoupling of state IV of oxidative phosphorylation by mutations in *Ranbp2* upon infectious agents and febrile states [33, 86] may espouse a predilection for higher glucose levels and enhanced anaerobic glycolysis upon insufficiency of RanBP2 and exposure to a variety of stressors.

Collectively, these and other data point toward a number of RanBP2-mediated metabolic and neurodegenerative diseases of acute and insidious clinical expressions sharing strong gene-environment interactions and broader spectra of metabolic footprints and pathomechanisms than hitherto realized. For example, PD and acute encephalopathies often result in striatal injury, but differ by the onset or progression of injury. Familial necrotic encephalopathies caused by mutations in *RANBP2* and a number of other encephalopathies of cerebral organic acid origin are often precipitated by episodes of febrile and infectious illnesses, which in turn may elicit fasting and catabolic stress, and secondarily, excitotoxic, and proinflammatory mechanisms [33, 87, 88]. Like other acute encephalopathies, infectious agents may also act as novel etiological agents in Parkinson disease [89, 90] and systemic metabolic deficits can directly promote dramatic clinical manifestations with distinct onsets and selective tissue expressions, such as age-dependent chorioretinal degenerations (e.g., gyrate atrophy) [91, 92], and once thought not to share pathomechanisms. In this regard, our findings strengthen the view that mouse models of *Ranbp2* are tractable genetic and physiological tools for the discovery of pathobiological mechanisms underlying diseases triggered by gene-environment interactions.

### Electronic supplementary material

Below is the link to the electronic supplementary material.

Supplementary material 1 (DOCX 53 kb)

Supplementary material 2 (pdf 1.70 MB)
