# Supplementary material for: Ranbp2 haploinsufficiency mediates distinct cellular and biochemical phenotypes in brain and retinal dopaminergic and glia cells elicited by the Parkinsonian neurotoxin, 1-methyl-4-phenyl-1,2,3,6-tetrahydropyridine (MPTP)
Source: Cell Mol Life Sci. 2012 Jul 21;69(20):3511–27. doi: 10.1007/s00018-012-1071-9 (PMC3445802; doi:10.1007/s00018-012-1071-9)
Supplement: Supplementary file 1 — Supplementary material 1 (DOCX 53 kb) [file 18_2012_1071_MOESM1_ESM.docx]

**Supplementary Information**

***Ranbp2* haploinsufficiency mediates distinct cellular and biochemical phenotypes in brain**

**and retinal dopaminergic and glia cells elicited by the Parkinsonian neurotoxin,**

**1-methyl-4-phenyl-1, 2, 3, 6-tetrahydropyridine (MPTP)**

Kyoung-in Cho, Kelly Searle, Mason Webb, Haiqing Yi and Paulo A. Ferreira

**Supplementary Materials and Methods**

***Immunohistochemistry reagents*.**

The primary antibodies used for immunohistochemistry in this study were polyclonal rabbit anti-TH antibody (1:500; Millipore/Pel-Freez Biologicals , Billerica), mouse anti-TH (1:100, Sigma-Aldrich, St Louis), rabbit anti-GFAP (1:250, DAKO, Glostrup), rat anti-mouse CD45 (BD Bioscience, Franklin Lakes), rabbit anti-CABP5 (1:100, gift from Dr. Haeseleer, University of Washington), rat anti-VMAT2 (1:100, Phoenix Pharmaceuticals, Berlingame), rat anti-glycine (1:50, gift from Dr. Pow, University of Queensland, Australia), rabbit anti-glutamine synthase (1:10,000, Sigma-Aldrich, St Louis), rat anti-mouse CD11b (1:50, Serotec, Oxford), goat anti-SOX2 (1:50, Santa Cruz, Santa Cruz). Alexa-conjugated secondary antibodies were from Invitrogen.

***Tissue collection* *for morphological and morphometric analyses.***

The eyeballs and brains were removed immediately from the skull after mice were killed. Small incisions were made on the anterior portion of eyeballs and these were fixed in 4% paraformaldehyde in phosphate-buffer saline (PBS), pH 7.4 for 3-hours (eyeballs), whereas brains were incubated for 48-hours. Brains were placed in a brain mould (Braintree, Chicago, IL) and the markers of the mouse brain atlas (<http://www.mbl.org/atlas170/atlas170_frame.html>) were used to section coronally with a razor blade at -2.40 to -3.80 from bregma for the collection of substantia nigra pars compacta (SNpc), ventral tegmental area (VTA) and periaqueductal gray area (PAGA), 0.74 to 0 mm from bregma for the collection of striatum, and -4.90 to -5.88 from bregma for the collection of the locus coeruleus (LC). Then, the collected brain sections were immersed in 5% agarose and sliced serially into 50 µm sections with a vibratome (Leica, Wetzlar). For radial cryosections of eyeballs, these underwent removal of the lens and were cryoprotected in 5% sucrose/100 mM PBS, pH 7.4, for 5 hours followed by 30% sucrose/100 mM PBS for 12 hours and then embedded in Tissue-Tek O.C.T. compound (Sakura Finetek, Torrence, CA). Radial cryosections 12 μm thick along the vertical meridian were collected and mounted on slides. For flat mounts of whole retinas, dissected eyeballs were fixed with 4% paraformaldehyde in phosphate buffered saline solution for 4 hrs at room temperature, retinas were removed, cut in a four-quadrant cloverleaf pattern using the caruncle as an orientation landmark and fixed for an additional 15 min in a 24-well plate. Whenever applicable, retinas were then incubated with primary antibody for 48 h at 4°C followed by incubation with Alexa Fluor secondary antibody (1:500; Invitrogen) for 4 hr at room temperature. Retinas were flattened on a glass slide with the ganglion cell layer side up and gently mounted on ProLong Gold antifade reagent (Invitrogen) with a cover slip. Images of immunolabeled cells of brain and retina were acquired with a Nikon C90i C1 Plus confocal scanning microscope controlled by the EZ-C1.3.10 confocal software (v6.4) and interfaced with 408-diode, 488/514-argon and 594 HeNe lasers.

Morphometric analyses of TH^+^amacrine and GFAP^+^-Müller cells was performed from eight and two 12 µm-thick sections, respectively, along the vertical meridian of the eyecup of each mouse. Quantitative measurement of gliosis was done by measuring the GFAP^+^-fluorescent intensity of confocal images from 150x100 μm areas of both edges (ora serrata) of the peripheral retina and 150x150 μm area of the central retina encompassing the optic nerve head. For retinal flat mounts, cell bodies of TH^+^-amacrine and EdU^+^-cells were tallied from three image fields of 860x860 μm each collected from a 50 μm depth of the inner retina with TH^+^-neurons serving as a midplane reference point. Cell bodies of TH^+^cells in the subtantia nigra (VTA and SNpc) and PAGA were counted from eighteen 50-µm-thick serial sections 100 µm apart of each hemisphere throughout the entire extent of these brains regions, whereas cell bodies of TH^+^cells throughout the entire extent of the LC region were counted from eighteen 50-µm-thick serial sections of each hemisphere. TH^+^-cells (~200) with nuclear atypia in the subtantia nigra were counted from 30 collapsed confocal *z*-stacks of images fields of 215x215x15 μm from 3 random coronal brain sections ranging bregma -2 to -3. Morphometric analyses and volume rendering were assisted by Metamorph V7.0 (Molecular Devices) and Nikon Elements AR (v3.2) software. *t*-test statistical analysis with two-tailed equal or unequal variance was performed. *p*≤0.05 was defined as significant.

***Overall mass spectrometry and analyses.***

At the time of analysis samples were extracted and prepared for analysis using Metabolon’s standard solvent extraction method as previously described (Evans *et al.* 2009, Lawton *et al.* 2008, Sreekumar *et al.* 2009). The extracted samples were split into equal parts for analysis on gas or liquid chromatography coupled to mass spectrometry (GC/MS and LC/MS/MS). For GC/MS, samples were analyzed on a Thermo-Finnigan Trace DSQ fast-scanning single-quadrupole mass spectrometer using electron impact ionization, whereas for LC/MS/MS samples were analyzed on a Waters ACQUITY UPLC and a Thermo-Finnigan LTQ mass spectrometer, which consisted of an electrospray ionization (ESI) source and linear ion-trap (LIT) mass analyzer.  Also included were several technical replicate samples created from a homogeneous pool containing a small amount of all study samples (“Client Matrix”). Compounds were identified by comparison to library entries of purified standards or recurrent unknown entities. Identification of known chemical entities was based on comparison to metabolomic library entries of purified standards. As of this work, more than 1000 commercially available purified standard compounds had been registered into a laboratory information management system (LIMS) for distribution to both the LC and GC platforms for determination of their analytical characteristics. The combination of chromatographic properties and mass spectra gave an indication of a match to the specific compound or an isobaric entity. Additional entities could be identified by virtue of their recurrent nature (both chromatographic and mass spectral).  Following log transformation and imputation with minimum observed values for each compound, a Welch’s two-sample *t*-test (assuming non-equal variances) was used to identify biochemicals/metabolites that differed significantly between the *Ranbp2^+/-^* treated and wild-type treated group. Biochemicals were sub-grouped into those that achieved statistical significance (*p*≤0.05) and those approaching significance (0.5< *p* <0.1).

**Sample Accessioning:** Each sample received was accessioned into the Metabolon LIMS system and was assigned by the LIMS a unique identifier, which was associated with the original source identifier only. This identifier was used to track all sample handling, tasks, results *etc.* The samples (and all derived aliquots) were bar-coded and tracked by the LIMS system. All portions of any sample were automatically assigned their own unique identifiers by the LIMS when a new task was created; the relationship of these samples was also tracked. All samples were maintained at -80 ºC until processed.

**Sample Preparation:** The sample preparation process was carried out using the automated MicroLab STAR® system from Hamilton Company. Recovery standards were added prior to the first step in the extraction process for QC purposes. Sample preparation was conducted using a proprietary series of organic and aqueous extractions to remove the protein fraction while allowing maximum recovery of small molecules. (See METHODS AND RESULTS section for study-specific methods.) The resulting extract was divided into two fractions; one for analysis by LC and one for analysis by GC. Samples were placed briefly on a TurboVap® (Zymark) to remove the organic solvent. Each sample was then frozen and dried under vacuum. Samples were then prepared for the appropriate instrument, either LC/MS or GC/MS.

**QA/QC:** For QA/QC purposes, a number of additional samples are included with each day’s analysis. Furthermore, a selection of QC compounds is added to every sample, including those under test. These compounds are carefully chosen so as not to interfere with the measurement of the endogenous compounds. Tables 1 and 2 describe the QC samples and compounds. These QC samples are primarily used to evaluate the process control for each study as well as aiding in the data curation.

**Table 1:** Description of Metabolon QC Samples

| **Type** | **Description** | **Purpose** |
| --- | --- | --- |
| MTRX | Large pool of human plasma maintained by Metabolon that has been characterized extensively. | Assure that all aspects of Metabolon process are operating within specifications. |
| CMTRX | Pool created by taking a small aliquot from every customer sample. | Assess the effect of a non-plasma matrix on the Metabolon process and distinguish biological variability from process variability. |
| PRCS | Aliquot of ultra-pure water | Process Blank used to assess the contribution to compound signals from the process. |
| SOLV | Aliquot of solvents used in extraction. | Solvent blank used to segregate contamination sources in the extraction. |

**Table 2:** Metabolon QC Standards

| **Type** | **Description** | **Purpose** |
| --- | --- | --- |
| DS | Derivatization Standard | Assess variability of derivatization for GC/MS samples. |
| IS | Internal Standard | Assess variability and performance of instrument. |
| RS | Recovery Standard | Assess variability and verify performance of extraction and instrumentation. |

**Liquid chromatography/Mass Spectrometry (LC/MS, LC/MS^2^**):  The LC/MS portion of the platform was based on a Waters ACQUITY UPLC and a Thermo-Finnigan LTQ mass spectrometer, which consisted of an electrospray ionization (ESI) source and linear ion-trap (LIT) mass analyzer.  The sample extract was split into two aliquots, dried, then reconstituted in acidic or basic LC-compatible solvents, each of which contained 11 or more injection standards at fixed concentrations. One aliquot was analyzed using acidic positive ion optimized conditions and the other using basic negative ion optimized conditions in two independent injections using separate dedicated columns. Extracts reconstituted in acidic conditions were gradient eluted using water and methanol both containing 0.1% Formic acid, while the basic extracts, which also used water/methanol, contained 6.5mM Ammonium Bicarbonate. The MS analysis alternated between MS and data-dependent MS^2^ scans using dynamic exclusion.

**Gas chromatography/Mass Spectrometry (GC/MS**):  The samples destined for GC/MS analysis were re-dried under vacuum desiccation for a minimum of 24 hours prior to being derivatized under dried nitrogen using bistrimethyl-silyl-triflouroacetamide (BSTFA).  The GC column was 5% phenyl and the temperature ramp is from 40° to 300° C in a 16 minute period.  Samples were analyzed on a Thermo-Finnigan Trace DSQ fast-scanning single-quadrupole mass spectrometer using electron impact ionization.  The instrument was tuned and calibrated for mass resolution and mass accuracy on a daily basis.  The information output from the raw data files was automatically extracted as discussed below.

**Accurate Mass Determination and MS/MS fragmentation (LC/MS**)**, (LC/MS/MS**):  The LC/MS portion of the platform was based on a Waters ACQUITY UPLC and a Thermo-Finnigan LTQ-FT mass spectrometer, which had a linear ion-trap (LIT) front end and a Fourier transform ion cyclotron resonance (FT-ICR) mass spectrometer backend.    For ions with counts greater than 2 million, an accurate mass measurement could be performed.  Accurate mass measurements could be made on the parent ion as well as fragments.  The typical mass error was less than 5 ppm.  Ions with less than two million counts require a greater amount of effort to characterize.  Fragmentation spectra (MS/MS) were typically generated in data dependent manner, but if necessary, targeted MS/MS could be employed, such as in the case of lower level signals.

**Bioinformatics:** The informatics system consisted of four major components, the Laboratory Information Management System (LIMS), the data extraction and peak-identification software, data processing tools for QC and compound identification, and a collection of information interpretation and visualization tools for use by data analysts. The hardware and software foundations for these informatics components were the LAN backbone, and a database server running Oracle 10.2.0.1 Enterprise Edition.

**LIMS:**  The purpose of the Metabolon LIMS system was to enable fully auditable laboratory automation through a secure, easy to use, and highly specialized system. The scope of the Metabolon LIMS system encompasses sample accessioning, sample preparation and instrumental analysis and reporting and advanced data analysis. All of the subsequent software systems are grounded in the LIMS data structures. It has been modified to leverage and interface with the in-house information extraction and data visualization systems, as well as third party instrumentation and data analysis software.

**Data Extraction and Quality Assurance:** The data extraction of the raw mass spec data files yielded information that could loaded into a relational database and manipulated without resorting to BLOB manipulation. Once in the database the information was examined and appropriate QC limits were imposed. Peaks were identified using Metabolon’s proprietary peak integration software, and component parts were stored in a separate and specifically designed complex data structure.

**Compound identification:** Compounds were identified by comparison to library entries of purified standards or recurrent unknown entities. Identification of known chemical entities was based on comparison to metabolomic library entries of purified standards. As of this writing, more than 1000 commercially available purified standard compounds had been acquired registered into LIMS for distribution to both the LC and GC platforms for determination of their analytical characteristics. The combination of chromatographic properties and mass spectra gave an indication of a match to the specific compound or an isobaric entity. Additional entities could be identified by virtue of their recurrent nature (both chromatographic and mass spectral). These compounds have the potential to be identified by future acquisition of a matching purified standard or by classical structural analysis.

**Curation:** A variety of curation procedures were carried out to ensure that a high quality data set was made available for statistical analysis and data interpretation. The QC and curation processes were designed to ensure accurate and consistent identification of true chemical entities, and to remove those representing system artifacts, mis-assignments, and background noise. Metabolon data analysts use proprietary visualization and interpretation software to confirm the consistency of peak identification among the various samples. Library matches for each compound were checked for each sample and corrected if necessary.

**Normalization:** For studies spanning multiple days, a data normalization step was performed to correct variation resulting from instrument inter-day tuning differences. Essentially, each compound was corrected in run-day blocks by registering the medians to equal one (1.00) and normalizing each data point proportionately (termed the “block correction”; Figure 1). For studies that did not require more than one day of analysis, no normalization is necessary, other than for purposes of data visualization.

**Figure 1:** Visualization of Data Normalization

**Statistical Calculation:** For many studies, two types of statistical analysis are usually performed: (1) significance tests and (2) classification analysis. (1) For pair-wise comparisons we typically perform Welch’s t-tests and/or Wilcoxon’s rank sum tests. For other statistical designs we may perform various ANOVA procedures (e.g., repeated measures ANOVA). (2) For classification we mainly use random forest analyses. Random forests give an estimate of how well we can classify *individuals* in a *new* data set into each group, in contrast to a t-test, which tests whether the unknown means for two populations are different or not. Random forests create a set of classification trees based on continual sampling of the experimental units and compounds. Then each observation is classified based on the majority votes from all the classification trees. Statistical analyses are performed with the program “R” <http://cran.r-project.org/>.

**References**.

Evans, A. M., DeHaven, C. D., Barrett, T., Mitchell, M. and Milgram, E. (2009) Integrated, nontargeted ultrahigh performance liquid chromatography/electrospray ionization tandem mass spectrometry platform for the identification and relative quantification of the small-molecule complement of biological systems. *Anal Chem,* **81,** 6656-6667.

Lawton, K. A., Berger, A., Mitchell, M. et al. (2008) Analysis of the adult human plasma metabolome. *Pharmacogenomics,* **9,** 383-397.

Sreekumar, A., Poisson, L. M., Rajendiran, T. M. et al. (2009) Metabolomic profiles delineate potential role for sarcosine in prostate cancer progression. *Nature,* **457,** 910-914.

**Supplementary Figure Legends**

**Supplementary figure 1**. MPTP does not cause remarkable changes of TH-immunostaining in the striatum between wild-type and *RANBP2^+/-^* mice. Confocal images of striatum regions of wild-type and *RANBP2^+/-^* mice at 6- and 14- days after MPTP treatment. Scale bars: 200 μm.

**Supplementary figure 2**. CD11b^+^-macrophages in the choroid plexus do not undergo proliferation upon MPTP treatment. There is a lack of EdU staining in CD11b^+^-macrophages (arrows) in retinal sections of mice provided with a daily EdU bolus for 13 days after MPTP treatment. Scale bars: 25 μm.
