# Supplementary figures and images for: Ranbp2 haploinsufficiency mediates distinct cellular and biochemical phenotypes in brain and retinal dopaminergic and glia cells elicited by the Parkinsonian neurotoxin, 1-methyl-4-phenyl-1,2,3,6-tetrahydropyridine (MPTP)
Source: Cell Mol Life Sci. 2012 Jul 21;69(20):3511–27. doi: 10.1007/s00018-012-1071-9 (PMC3445802; doi:10.1007/s00018-012-1071-9)

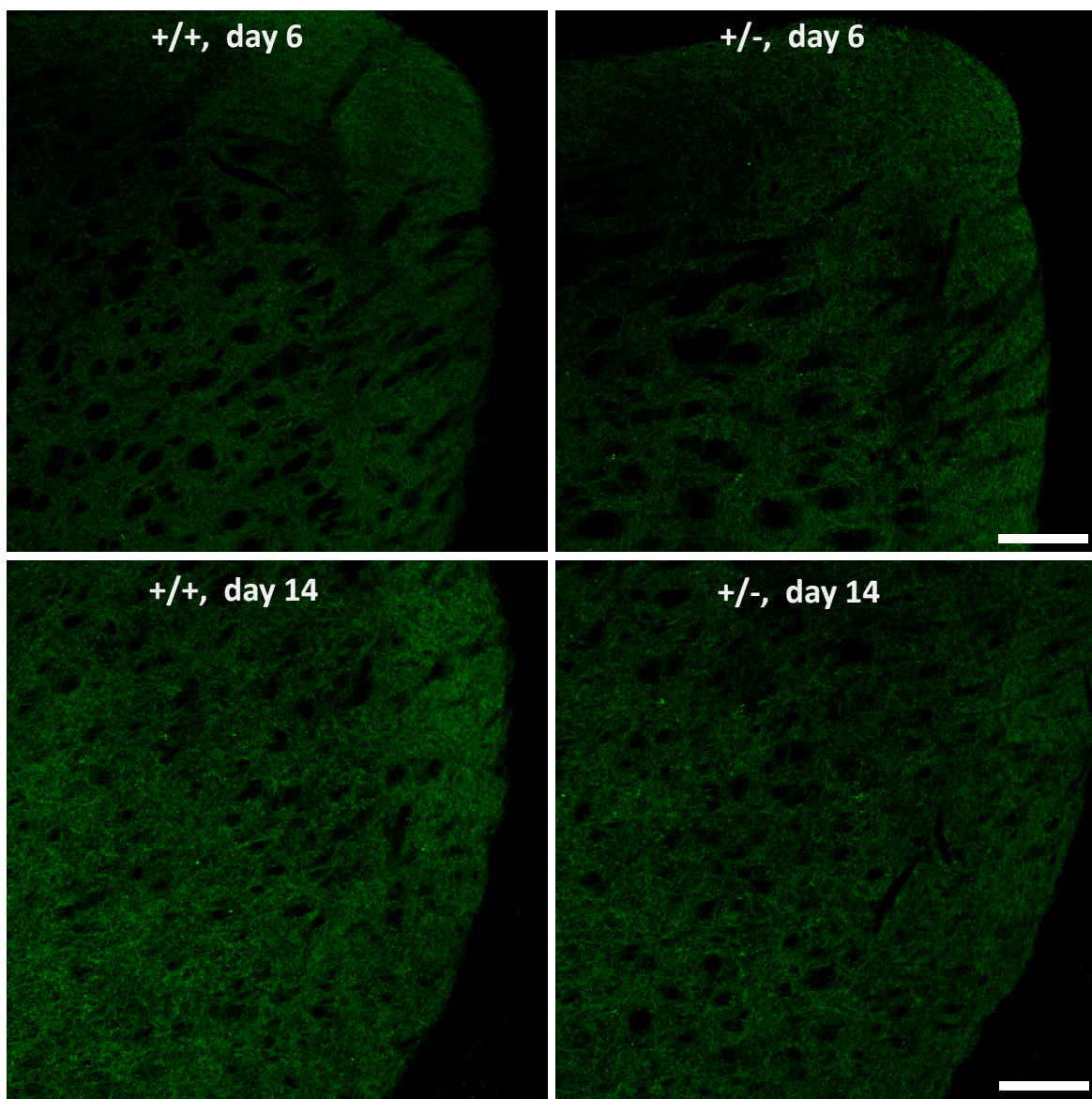

Supplementary Figure 1

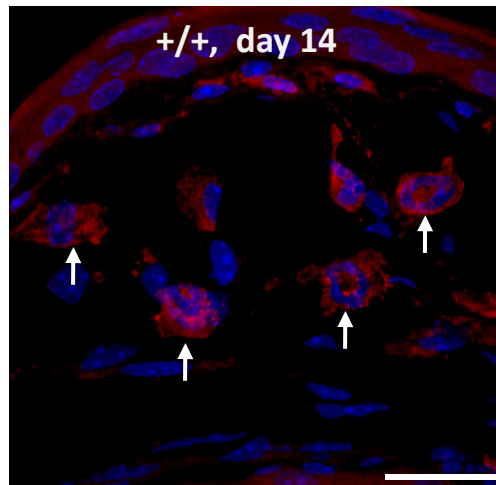

Supplementary Figure 2

Supplement: Supplementary file 2 — Supplementary material 2 (pdf 1.70 MB) [file 18_2012_1071_MOESM2_ESM.pdf]
